# DHPA-Containing Cobalt-Based Redox Metal-Organic Cyclohelicates as Enzymatic Molecular Flasks for Light-Driven H_2_ Production

**DOI:** 10.1038/s41598-017-14728-8

**Published:** 2017-10-30

**Authors:** Liang Zhao, Jian Wang, Pengyan Wu, Cheng He, Xiangyang Guo, Chunying Duan

**Affiliations:** State Key Laboratory of Fine Chemicals, Dalian University of Technology, Dalian, 116023 P. R. China

## Abstract

The supramolecular assembly of predesigned organic and inorganic building blocks is an excellent tool for constructing well-defined nanosized molecular cavities that catalyse specific chemical transformations. By incorporating a reduced nicotinamide adenine dinucleotide (NADH) mimic within the ligand backbone, a redox-active cobalt-based macrocycle was developed as a redox vehicle for the construction of an artificial photosynthesis (AP) system. The cyclohelicate can encapsulate fluorescein within its cavity for light-driven H_2_ evolution, with the turnover number (TON) and turnover frequency (TOF) reaching 400 and 100 moles H_2_ per mole redox catalyst per hour, respectively. Control experiments demonstrated that the reactions were potentially occurred within the cavity of the cyclohelicates which were inhibited in the presence of adenosine triphosphate (ATP), and the redox-active NADH mimic dihydropyridine amido moieties within the ligands played an important role in photocatalytic proton reduction process.

## Introduction

Metal-organic macrocycles represent a unique class of functional molecular containers that display interesting recognition properties and fascinating reactivity similar to natural enzymes^1,2^. The architectures generating well-defined cavities provided specific inner environments for the selective bonding of guest molecules and catalysing their reactions^3,4^. Inspired by the pocket feature of natural enzymes, functional coordination cages with various structures and catalytic activities have been developed to achieve the excellent catalytic ability of natural enzymes^5–7^. However, few Werner-type capsules have been used as mimics of highly evolved and finely tuned molecular photosynthetic systems^8,9^, despite their importance in living systems and sustainable solar energy conversion^10–12^. As artificial photosynthesis (AP) systems always involve a photosensitizer for light absorption, a catalyst for H_2_ generation, and an electron donor^13,14^, the intrinsic difficulties in mimicking photosynthetic systems include the feasibility of processing in an aqueous solution and a well-defined cavity to bring the proton reduction centre and photosensitizer closer together^15,16^. Most importantly, metal-organic nanocages should comprise at least one of the two basic functional units that exhibit redox activity and/or light-harvesting ability for the possible construction of highly efficient and easily operated supramolecular AP systems^17,18^.

Reduced nicotinamide adenine dinucleotide (NADH) plays an important role in the reduction-oxidation metabolism of some most important coenzymes found in living cells^19–21^. The dihydropyridine amido (DHPA) group is described as the key structure in NADH models^22^ and an important part in electron transfer. Therefore, the incorporation of a DHPA group into the ligand backbone as the active site should be a powerful approach to mimic the activity of these enzymes *i*.*e*., the cofactor in [FeFe]-hydrogenase that controls the redox levels by sharing the effect of electron gain, loss and distribution^23–25^.

**Figure 1:**
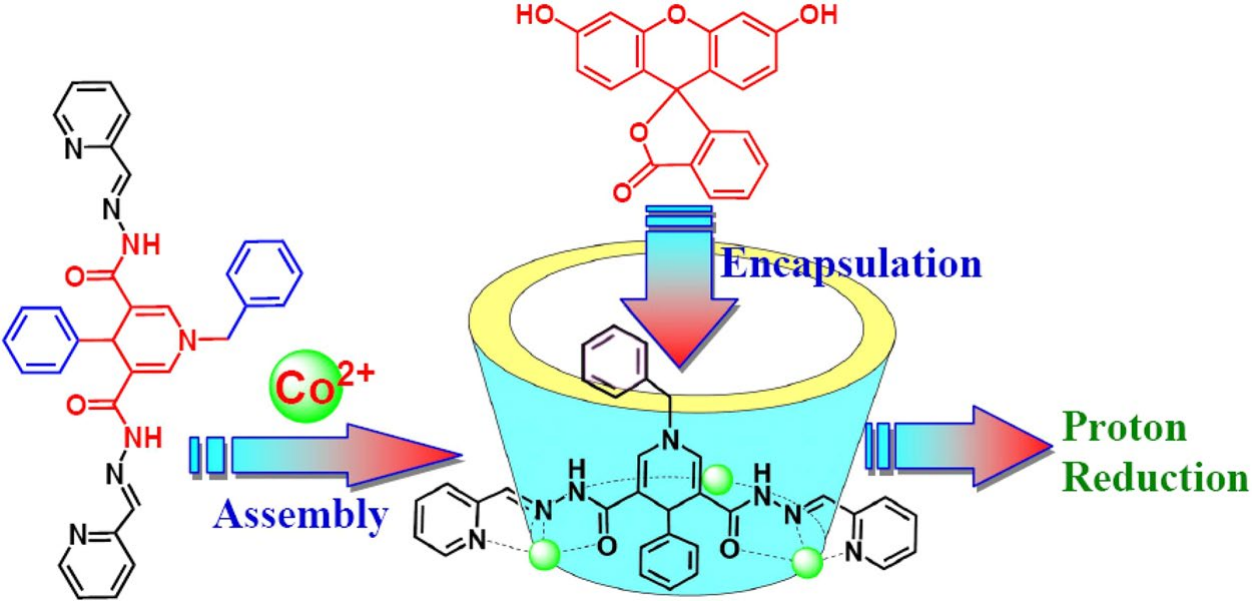
Perspective view of the supramolecular structure of the AP system for light-driven H_2_ production, showing the construction of the nanocage host as a proton reductive catalyst and the encapsulation of **Fl** guest as a photocatalyst.

Through modulation of tridentate N_2_O units containing amide groups on a central dihydropyridine ring at the *meta* sites (Fig. 1), we developed new cobalt-based redox-active helical triangles to encapsulate a photosensitizer for light-driven H_2_ production. We reasoned that the amide groups in the positively charged cages matched the functional requirements and could offer hydrogen bonding interactions for the recognition of fluorescein (**Fl**). The confinement of the cavity possibly enforced the proximity between the redox-active cobalt(II) centres and **Fl**, enhancing the PET efficiency to avoid unwanted energy transfer or reverse-ET reactions^26,27^. The mild redox couple of DHPA close to the H_2_/H^+^ couple and the geometric position of DHPA close to the redox catalyst centre made this supramolecular system a more complete working model of AP systems^28,29^. Control experiments based on the reference compound that has the similar structural feature and almost same coordination geometries, as well as redox potential with that of the original, but without the DHPA fragments were also carried out for a comparison.

## Results and Discussion

**Figure 2:**
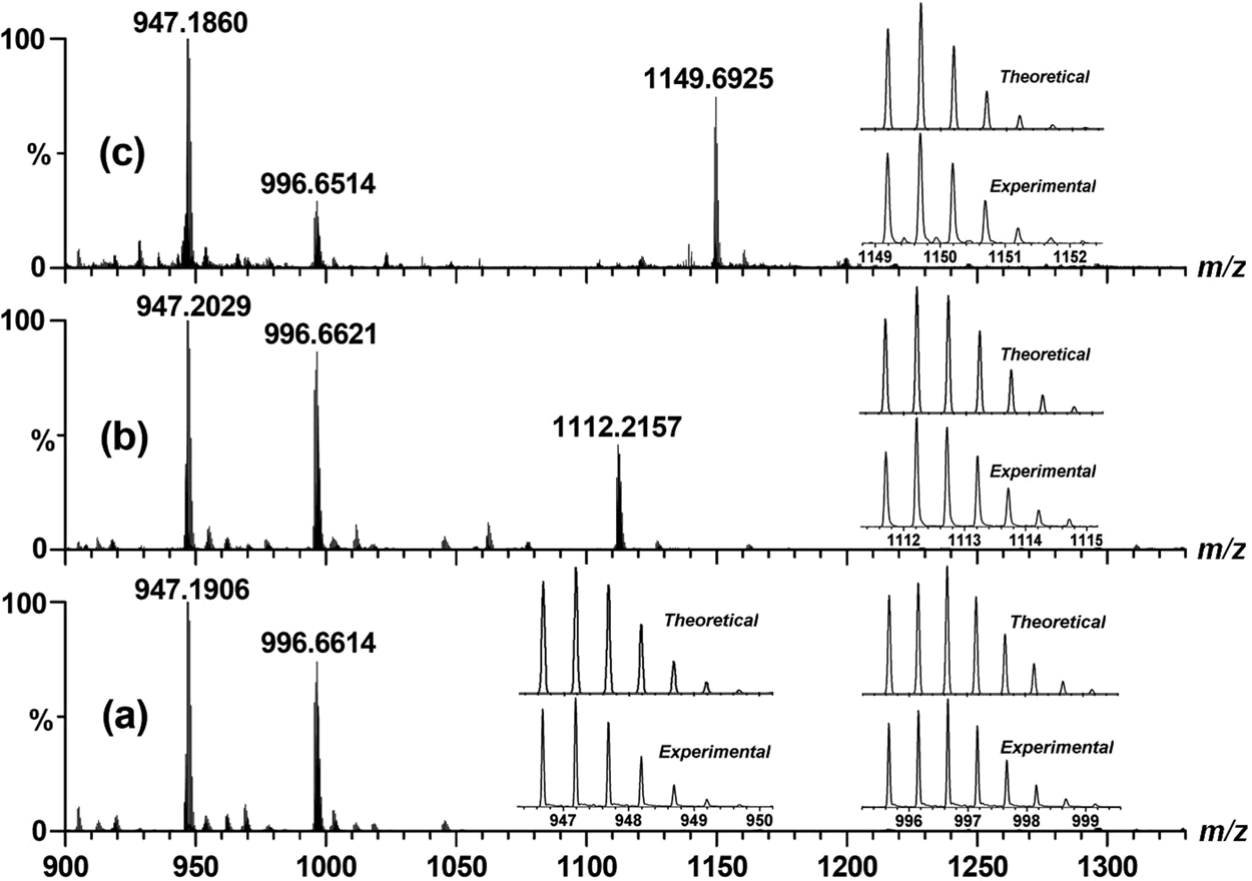
ESI-MS of (**a**) Co–**ZPB** (0.1 mM) and (**b**) Co–**ZPB** (0.1 mM) after the addition of 1 equiv. of **Fl** in CH_3_CN solution. ESI-MS of (**c**) Co–**ZPB** (0.1 mM) after the addition of 1 equiv. of ATP in CH_3_CN/H_2_O solution. Insets show the measured and simulated isotopic patterns at 947.19, 996.66 (**a**), 1112.22 (**b**) and 1149.69 (**c**).

The ligand H_2_ **ZPB** contianing two tridentate coordinated units was obtained from the reaction of 2-pyridyl aldehyde with malono-hydrazide in an ethanol solution. Evaporating a solution containing equivalent molar ratios of H_2_ **ZPB** and Co(NO_3_)_2_·6H_2_O in the presence of NaClO_4_ for several days led to the formation of the compound Co–**ZPB**. ESI-MS spectrum of the formed Co–**ZPB** solid exhibited intense peaks at *m*/*z* = 947.19 and *m*/*z* = 996.66, with the isotopic distribution patterns separated by 0.50 ± 0.01 Da, and a comparison with the simulation results based on natural isotopic abundances suggested that these peaks are assigned to [Co_3_(H**ZPB**)_3_·ClO_4_]^2+^ and [Co_3_(H**ZPB**)_2_(H_2_ **ZPB**) **·**2ClO_4_]^2+^, respectively, indicating the successful assembly of a Co-based M_3_L_3_ molecular macrocycle (Fig. 2a). Tridentate (N_2_O) coordinated units sharing two five-membered chelating rings are one kind of efficient building blocks that have been widely used to construct stable and functional discrete architectures with regular structure and high symmetry^30,31^. According to our previous work, each of three cobalt centres typically coordinated with two planar tridentate N_2_O chelators to form a *mer* configuration molecular macrocycle with considerable stabilities. In the presence of **Fl**, the ESI-MS spectrum of Co–**ZPB** exhibited a new peak at *m*/*z* = 1112.22 that was assigned to [Co_3_(H**ZPB**)_3_·ClO_4_ ⊃ **Fl**]^2+^ through comparison with the simulation results obtained based on natural isotopic abundances (Fig. 2b), which indicates the ability of Co–**ZPB** to encapsulate **Fl** within its cavity.

^1^H NMR spectrum of Co–**ZPB** recorded after the addition of a 1.0 molar ratio of **Fl** exhibited significant upfield shifts of protons H_3,6_ (δ = 0.15 ppm) and other protons, suggesting that **Fl** was encapsulated within the electron-rich cavity of Co–**ZPB** (Figure S5 in supporting information). UV-Vis titration of Co–**ZPB** upon addition of **Fl** caused a significant absorption enhancement at 510 nm. The titration curve of this band reflected the formation of 1:1 stoichiometric ratio of the host-guest complexation, with a calculated association constant of 2.19 × 10^5^ M^−1^ (Figure S9 in supporting information)^32^. It is postulated that the amide groups located within the positively charged macrocycle introduced geometric and functional properties that are beneficial to the recognition of the organic dye^33,34^.

**Figure 3:**
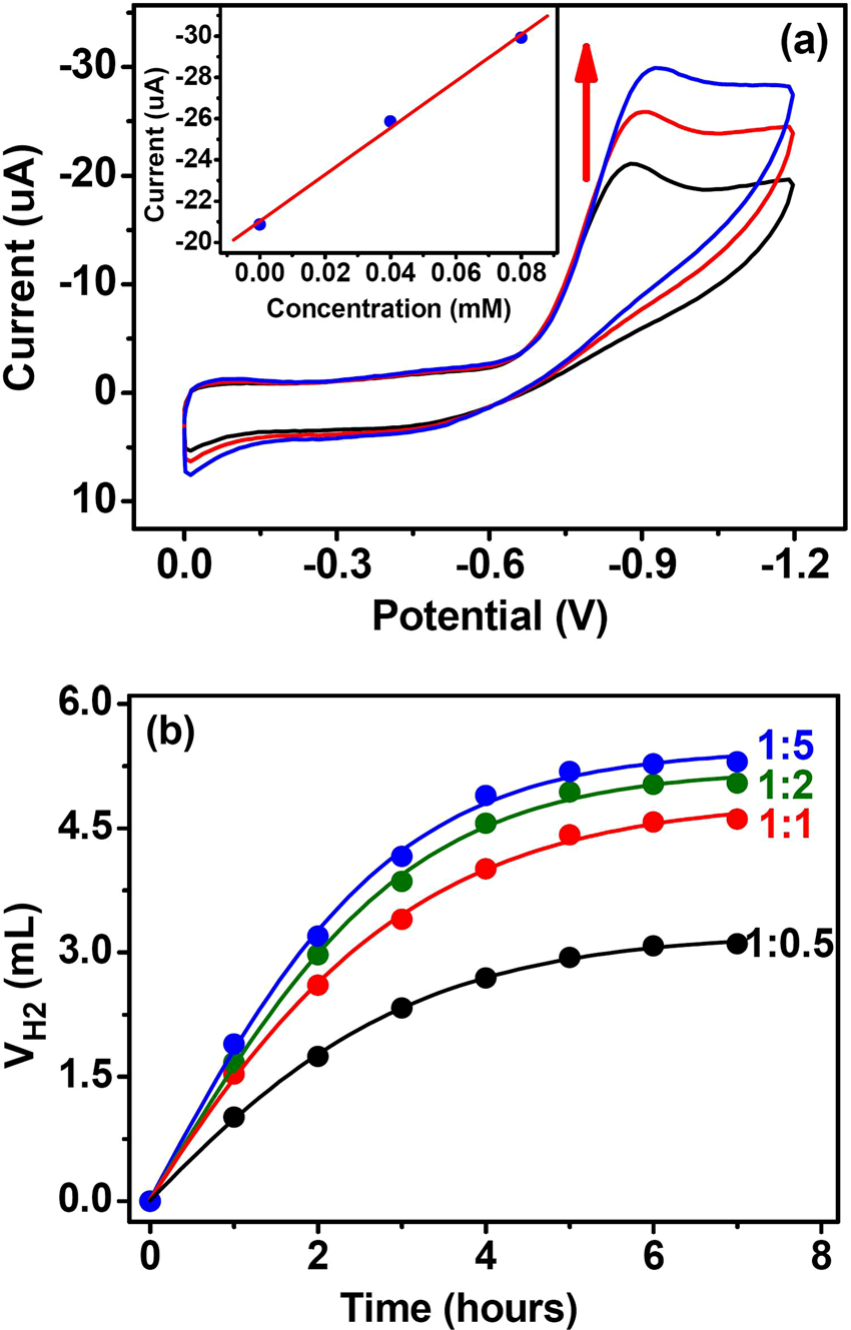
(**a**) Cyclic voltammograms of Co–**ZPB** (0.1 mM) containing *n*-Bu_4_NClO_4_ (0.1 M) in a CH_3_CN solution and upon the addition of Et_3_NHCl with concentrations of 0.04 mM (red line) and 0.08 mM (blue line), respectively. Scan Rate: 100 mV/s. The inset showing the *i*_c_ *vs*. [HNEt_3_]. (**b**) H_2_ production after irradiation of the system containing TEA (5%), Co–**ZPB** (0.1 mM) and different concentrations of **Fl**.

**Figure 4:**
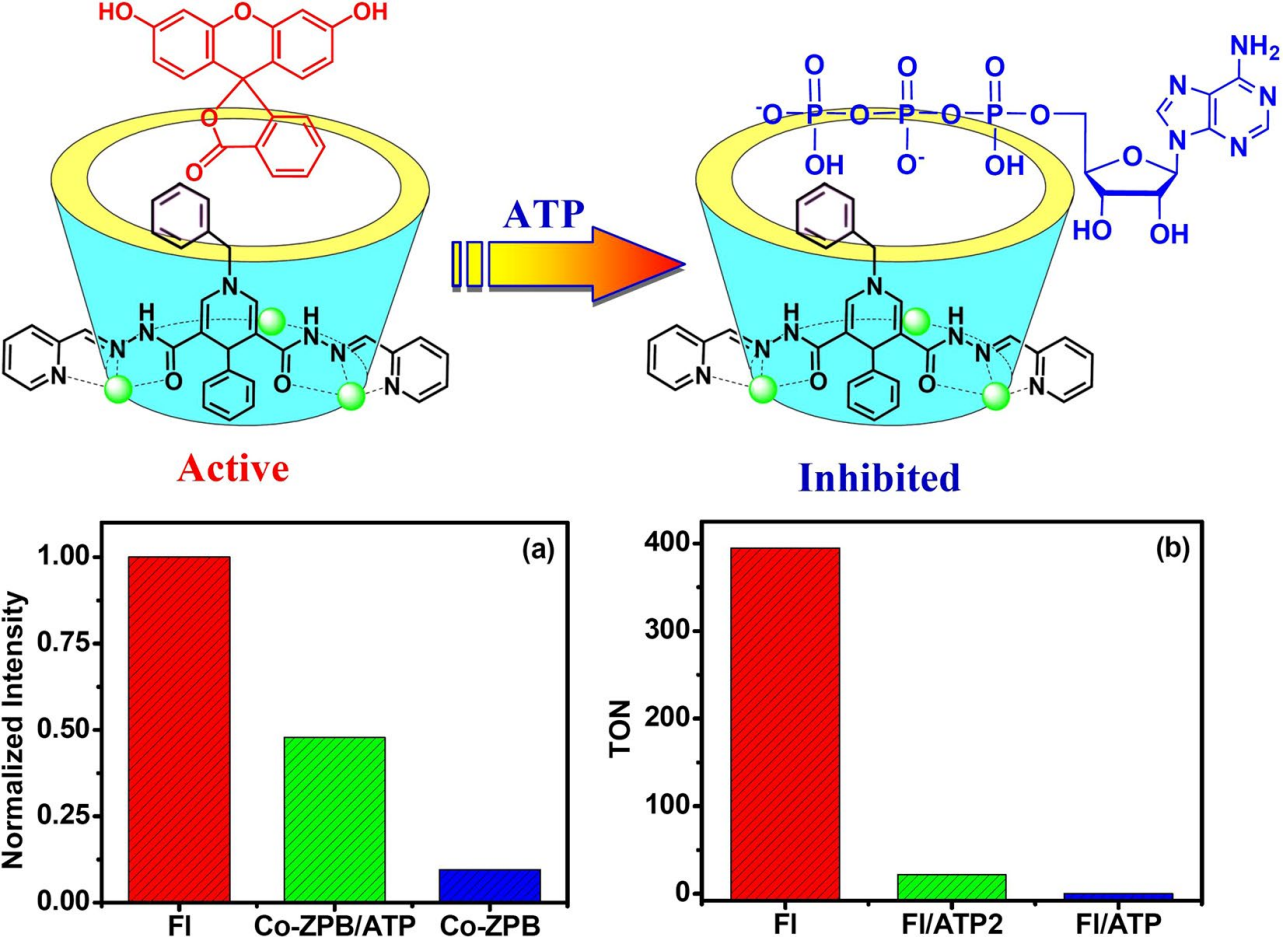
(**a**) Normalized fluorescence of **Fl** (0.01 mM) (red bar) in CH_3_CN/H_2_O (1:1) at pH 11.0 in the presence of Co–**ZPB** (0.015 mM) (blue bar) and in the presence of Co–**ZPB** (0.015 mM) and ATP (0.15 mM) (green bar). The intensities were recorded at 520 nm, with excitation at 460 nm. (**b**) TON of photocatalytic H_2_ evolution after 6 hours of irradiation in CH_3_CN/H_2_O (1:1) at pH 11.0 with 5% TEA for the system containing Co–**ZPB** (0.1 mM)/**Fl** (0.1 mM) (red bar) in the presence of 0.2 mM ATP (green bar) or 0.2 mM ATP (blue bar).

The cyclic voltammogram of Co–**ZPB** in CH_3_CN exhibited broad peak at −0.88 V (vs. Ag/AgCl). Because redox potentials of the cobalt centers with same coordination environment in compound Co–**QDB** (*vide infra*) and of the DHPA moiety are very close to this value (Figures S20 and S21 in supporting information), the peak was assigned to the overlap of Co^II^/Co^I^ reduction reaction with the reduction reaction of the DHPA moiety. Clearly, Co–**ZPB** is well suited to explore the redox-induced reactions that occur near the H_2_/H^+^ couple (Fig. 3a). When the addition of increasing amounts of Et_3_NH^+^ triggered the appearance of a new irreversible cathodic wave near the Co^II^/Co^I^ response. Increasing the acid concentration raised the height of the new wave with a linear relationship and shifted it to more negative potentials. The new wave was assigned to proton reduction, suggesting that Co–**ZPB** can reduce protons in a catalytic reaction^35,36^. Moreover, as the oxidation potential of **Fl** in its photoexcited state (FI* → FI^+^ + e^−^) and ground state (**Fl** → **Fl** ^+^ + e^−^) are −1.55 V and 0.87 V (vs SCE)^37^, respectively, the photoexcited state of **Fl** (Fl*) has sufficient capability to reduce Co(II) to Co(I) directly. In the meantime, Co–**ZPB** was also an efficient quencher of the photosensitizer **Fl** (Fig. 4a and Figure S12 in supporting information). The addition of Co–**ZPB** to the solution of **Fl** (10 *μ*M) in 1:1 CH_3_CN/H_2_O caused significant emission quenching. The quenching behaviour is considered a photoinduced electron transfer process from the excited state of **Fl** (Fl*) to Co–**ZPB**, enabling the activation of Co–**ZPB** by **Fl** for H_2_ production in solution^38,39^.

The photocatalytic activities of Co–**ZPB** (0.1 mM) assembled with **Fl** (0.1 mM) towards evolution of molecular hydrogen were evaluated in an acetonitrile/water solution at room temperature in the presence of 5% triethylamine (TEA) as the sacrificial electron donor^40,41^. The volume of H_2_ was quantified at the end of the photolysis by GC of the headspace gases. Our system could work at pH range from 10.5 to 12.5, with maximal H_2_ evolution at pH 11.0 (Figure S15 in supporting information). The initial calculated turnover frequency (TOF) was approximately 100 moles H_2_ per mole catalyst per hour, with a turnover number (TON) of approximately 400 moles H_2_ per mole of catalyst (Fig. 3b). Notably, the TON for **Fl** and the redox catalyst was obtained in a stoichiometric catalyst/photosensitizer ratio. Compared to the intermolecular systems in which the TON value of one component is optimized with the other component in greater excess, the TON in the stoichiometric system reflects the true activity of the AP system. Meantime, ESI-MS spectrum of the Co−**ZPB** after reaction exhibited intense peaks at *m*/*z* = 947.21, 1062.71 and 1112.20, with the isotopic distribution patterns separated by 0.50 ± 0.01 Da. The peaks were assigned to host and host-guest complex, respectively, indicating the Co−**ZPB/Fl** system has sufficient structural stabilities during the reaction (Figure S2 in supporting information).

At a fixed **Fl** concentration (0.1 mM), the initial rates of H_2_ generation increased with the [Co–**ZPB**] at lower concentrations (<0.1 mM) (Figure S19 in supporting information). When [Co–**ZPB**] was fixed 0.1 mM and the **Fl** concentration was varied, the TOF plateaued at 0.1 mM; further addition of **Fl** did not increase the lifetime or TON of Co–**ZPB**. In all cases, the optimal conditions consisted of a constant molar ratio of Co–**ZPB**/**Fl**. An increase in the Co–**ZPB**/**Fl** ratio decreased the TON, and a decrease in the Co–**ZPB**/**Fl** ratio hardly increased the TONs of **Fl** or Co–**ZPB**. A 1:1 stoichiometric ratio of Co–**ZPB**/**Fl** complexation species apparently dominated the photosynthetic system. Control experiments demonstrated that **Fl**, Co–**ZPB** and light are essential for H_2_ generation.

To confirm whether the photoinduced H_2_ production occurred within the cavity of Co–**ZPB** or through a normal homogeneous system, the photocatalytic reaction was inhibited by the addition of a non-reactive species, adenosine triphosphate (ATP), to the reaction mixture because previous work showed that a cobalt-based cyclohelicate recognized ATP^42^. As expected, the presence of the molecular host Co–**ZPB** led to obvious upfield shifts of the aromatic protons on the adenosine ring, suggesting that ATP was encapsulated within the cavity of the macrocyclic complex (Figure S6 in supporting information). The ESI-MS spectrum of Co–**ZPB** in the presence of ATP exhibited an intense peak at *m*/*z* = 1149.69, with the isotopic distribution patterns separated by 0.50 ± 0.02 Da. This peak was assigned to [Co_3_(H**ZPB**)_2_(H_2_ **ZPB**) ⊃ ATP]^2+^, indicating the stable existence of Co–**ZPB** in solution and the successful encapsulation of ATP within the cavity of Co–**ZPB** (Fig. 2c).

Importantly, the addition of ATP to replace the photosensitizer or redox catalyst Co–**ZPB** did not result in any H_2_ production, but the presence of 0.3 mM ATP effectively stopped the photocatalytic H_2_ production of the Co–**ZPB** (0.1 mM)/**Fl** (0.1 mM) system (Fig. 4b). This competitive inhibition behaviour was described as enzymatic-like and suggested that the H_2_ production possibly occurred within the cavity of Co–**ZPB**. The UV-Vis titration of Co–**ZPB** after the addition of ATP caused a significant decrease in absorption at 510 nm. The titration curve confirmed the 1:1 stoichiometric host-guest behaviour with an association constant of 3.64 × 10^6^ M^−1^ (Figure S17 in supporting information). This value was thirty fold larger than that of the encapsulation of **Fl**, demonstrating the possibility of ATP to substitute for **Fl** to encapsulate the cavity of the metallohelicate. The H_2_ production likely occurred within the cavity of Co–**ZPB**, rather than in a normal homogeneous system^2,43^.

**Figure 5:**
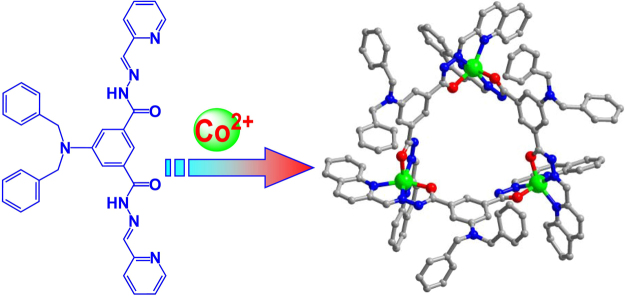
Procedure for the synthesis and crystal structure of the cobalt-based cyclohelicate showing the coordinated geometry of the redox-active metal centre. Solvent molecules and anions are omitted for clarity. The metal, oxygen, nitrogen and carbon atoms are drawn in green, red, blue and grey respectively.

The incorporation of a DHPA group into the ligand backbone as the active site seemed being a powerful approach to adjust the overpotential of the metal sites for proton reduction by sharing the effect of electron gain, loss and distribution. To further investigate the important role of the NADH model in the proton reduction process, a new metallohelicate Co–**QDB** that has the similar molecular structural features and coordination geometries of cobalt centers with that of Co–**ZPB**, but without fragment of the DHPA group was synthesized and structurally characterized for comparison. The ligand H_2_ **QDB** was synthesized through the reaction of 2-quiolinecarboxaldehyde with 5-(dibenzylamino)isophthalohydrazide according to the literature method (Fig. 5)^44^. Co–**QDB** was prepared in a yield of 75% by layering a methanol solution of Co(NO_3_)_2_·6H_2_O onto a dichloromethane solution of H_2_ **QDB** in the presence of NH_4_PF_6_. The ESI-MS spectrum of Co–**QDB** exhibited intense peaks at *m*/*z* = 1088.05, 1119.55 and 1161.03, with the isotopic distribution patterns separated by 0.5 ± 0.01 Da, and a comparison with the simulation results based on natural isotopic abundances suggested that the peaks are assigned to [Co_3_(**QDB**) (H**QDB**)_2_]^2+^, [Co_3_(H**QDB**)_3_(NO_3_)]^2+^ and [Co_3_(H**QDB**)_3_(PF_6_)]^2+^, respectively, revealing the same structure and stability of the Co–**QDB** in solution (Figure S3 in supporting information). Single-crystal X-ray analysis confirmed the formation of a pseudo-*C*_3_ symmetric macrocyclic helicate with three cobalt ions and three deprotonated H**QDB** ligands connected in an alternating pattern (Figure S1 in supporting information). Each cobalt centre was coordinated by two tridentate N_2_O chelating groups in a mer geometry with pairs of O atoms and amide N atoms each bearing a *cis* relationship, whereas the acetohydrazide N atoms were *trans* to each other that further indicate the *mer* configuration of the Co–**ZPB**. The measured C–O, C–N and N–N bond distances were all within the normal range of single and double bonds, pointing to the extensive electron delocalization over the entire molecular skeleton (Table S1 in supporting information)^45,46^. The separations between cobalt ions were 9.58 Å on average, and the average separation between the tertiary amine N atoms was 11.36 Å. The presence of four counter anions revealed that only two of the amide groups lost their protons during the coordination. These amide groups provided geometric and functional properties beneficial to the recognition of organic dyes, as observed in our previous works.

**Figure 6:**
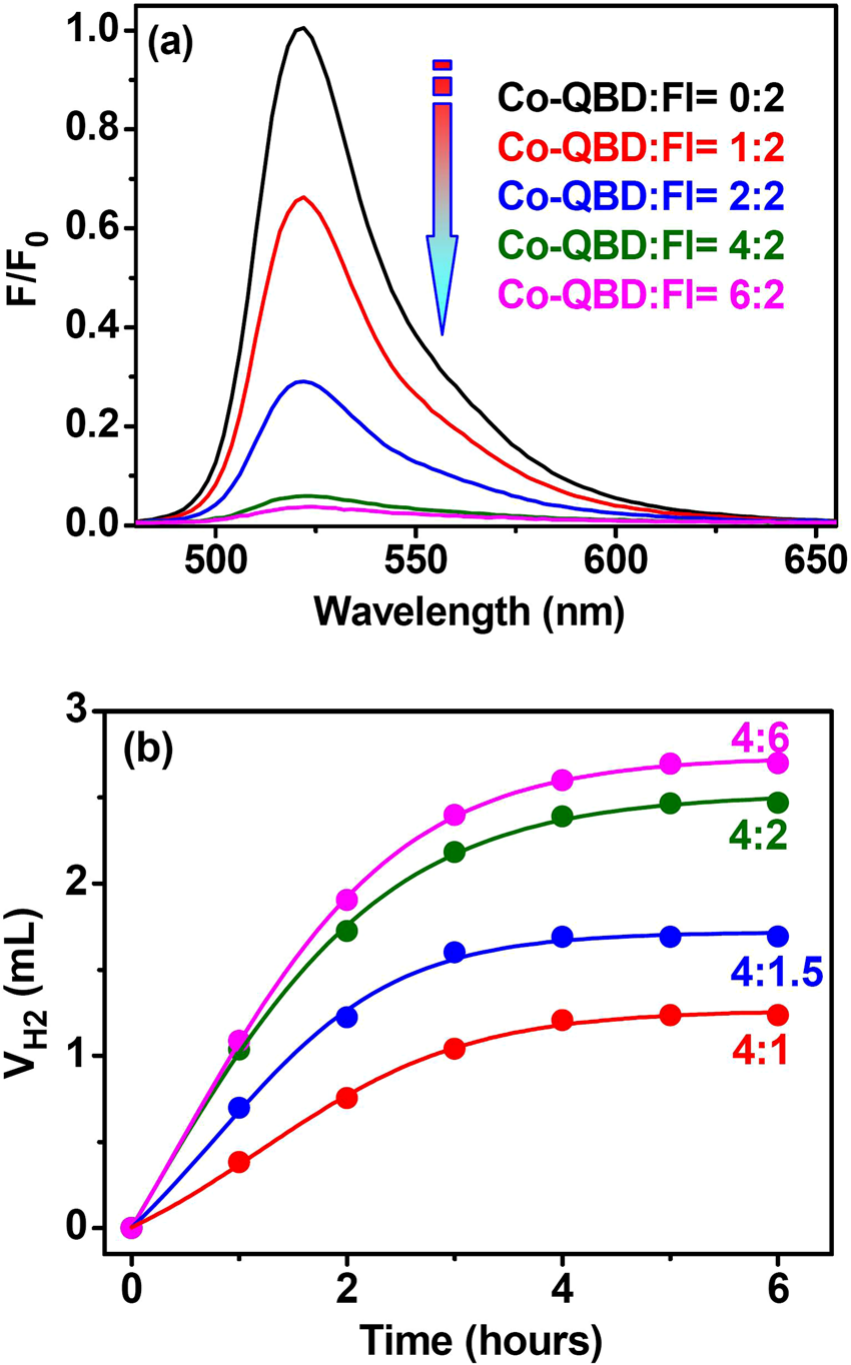
(**a**) Family of the emission spectra of **Fl** (0.01 mM) in the presence of TEA in 1:1 CH_3_CN/H_2_O at pH 11.0 after the addition of different concentrations of Co–**QDB**. (**b**) H_2_ production after irradiation the system containing TEA (5%), Co–**QDB** (80 μM) and different concentrations of **Fl**.

The cyclic voltammogram of Co–**QDB** recorded in DMF exhibited one reversible reduction of Co^II^/Co^I^ at −1.08 V (vs. Ag/AgCl). This potential falls well within the redox range of reducing a proton in aqueous media^47^, enabling the host to be a redox catalyst for proton reduction (Figure S21 in supporting information). Co–**QDB** was also demonstrated to be an efficient quencher of the excited state of **Fl** through photoinduced electron transfer (Fig. 6a and Figure S14 in supporting information). Photolysis of a solution of 0.04 mM **Fl** and 0.08 mM Co–**QDB** in a solvent mixture containing TEA (5% v:v) in DMF/CH_3_CN/H_2_O resulted in H_2_ generation, with optimal photocatalysis at pH 10.0 (Figure S16 in supporting information). As shown in Fig. 6b, the initial TOF was approximately 40 moles H_2_ per mole catalyst per hour, with a TON of approximately 250 moles H_2_ per mole of catalyst. The TON and TOF of the Co–**QDB**/**Fl** system is obviously lower than those of the Co–**ZPB**/**Fl** system.

Interestingly, the Co–**QDB**/**Fl** ratio is crucial: the TON plateaus at a 2:1 stoichiometric ratio of Co–**QDB**/**Fl** under the optimal conditions. At a fixed Co–**QDB** concentration (0.08 mM), the decrease in the Co–**QDB**/**Fl** ratio decreased the TON, and the increase in the Co–**QDB**/**Fl** ratio hardly increased the TON of **Fl** or Co–**QDB**, suggesting that a potential 2:1 stoichiometric ratio of the Co–**QDB**/**Fl** complexation species dominated the photosynthetic system (Fig. 6b). Additionally, glutathione (GSH), an important compound in natural systems that is inactive toward hydrogenation, was chosen as an inhibitor because our previous work showed that an isostructural cyclohelicate could recognize GSH well^44^. When the addition of 0.2 mM GSH to the 2:1 Co–**QDB** (0.08 mM)/**Fl** (0.04 mM) system directly stopped the photocatalytic H_2_ production. Since GSH does not exhibit any suitable redox potential for H_2_ production, this competitive inhibition suggested demonstrated that the H_2_ production occurred within the cavity of Co–**QDB**.

The ESI-MS spectrum of Co–**QDB** in the presence of **Fl** exhibited intense peaks at *m*/*z* ~2360.27 assigned to {K[Co_3_(H**QDB**)(**QDB**)_2_]_2_ ⊃ **Fl**}^2+^, providing additional proof for the 2:1 stoichiometric complexation behaviour (Figure S3 in supporting information). After irradiating the system for 6 hours, ESI-MS spectrum of the Co−**QDB** also exhibited intense peaks at *m*/*z* = 1088.05, 1161.03, and 2341.27, with the isotopic distribution patterns separated by 0.50 ± 0.01 Da. The peaks are assigned to host and host-guest complex, respectively, indicating the Co−**QDB** system also has sufficient structural stabilities in the reaction process (Figure S4 in supporting information). UV-Vis titration of Co–**QDB** after addition of **Fl** supported the 2:1 stoichiometry of the host-guest complexation, with an association constant of 8.32 × 10^9^ M^−2^ (Figure S10 in supporting information). The ^1^H NMR spectrum of Co–**QDB** after the addition of a 0.5 molar ratio of **Fl** exhibited significant upfield shifts of protons H_3,6_ (δ = 0.13 *ppm*) and other protons, reflecting the encapsulation of **Fl** within the cavity of the macrocycle Co–**QDB** (Figure S7 in supporting information). Of course Co–**QDB** was able to recognize GSH in similar aqueous media (Figure S8 in supporting information). UV-Vis absorption titration of Co–**QDB** after the addition of GSH also induced quenching and suggested the formation of a 1:1 stoichiometry of the host–guest complexation with an association constant of 5.05 × 10^5^ M^−1^ (Figure S18 in supporting information). At a fixed Co–**QDB** concentration of 0.08 mM, the presence of GSH could substitute for **Fl** to occupy the cavity of Co–**QDB**. It is hypothesized that Co–**ZPB** and Co–**QDB** are true molecular flasks^48,49^, within which AP systems are assembled through encapsulation of an organic dye as a photosensitizer.

## Conclusion

In summary, we have reported the preparation of a redox-active cobalt-based macrocycle through the incorporation of an NADH mimic within the ligand backbone and a new strategy for the construction of AP systems. The metal-organic cyclohelicate is an enzymatic molecular flask and encapsulated **Fl** within its cavity for light-driven H_2_ evolution with a TON and TOF that reached 400 and 100 moles H_2_ per mole redox catalyst per hour, respectively. The reaction was inhibited by the presence of ATP and occurred within the cavity of the cyclohelicate. The control experiments indicated that the redox-active dihydropyridine amido group of the NADH mimic was helpful for the photocatalytic proton reduction process. By incorporating other redox-active or photoactive functional groups, this strategy can be extended to highly active AP systems.

## Materials and Methods

### Materials

All chemicals were reagent grade, obtained from commercial sources and used without further purification. The elemental analyses of C, H and N were performed on a Vario EL III elemental analyser. ^1^H NMR spectra were measured on a Varian INOVA 400 M spectrometer. ESI mass spectra were obtained on an HPLC-Q-TOFMS instrument using methanol as the mobile phase. UV-Vis spectra were measured on an HP 8453 spectrometer. The solution fluorescence spectra were obtained using an FLS920 spectrometer (Edinburgh Instruments). Both the excitation and emission slit widths were 2 nm. The solutions of Co–**ZPB** (1.0 × 10^−3^ M) and Co–**QDB** (4.0 × 10^−3^ M) were prepared in CH_3_CN and DMF, respectively. Stock solutions of **Fl** (1.0 × 10^−3^ M) were prepared directly in CH_3_CN and were excited at 460 nm.

All electrochemical measurements were carried under nitrogen at room temperature on a CHI 1130 (CH Instrument Co., Shanghai) electrochemical analyser with a conventional three-electrode system consisting of a homemade Ag/AgCl electrode as the reference electrode, a platinum silk electrode with a 0.5 mM diameter as the counter electrode, and a glassy carbon electrode as the working electrode. Cyclic voltammograms were recorded at solution concentrations of 0.1 mM and 1.0 mM for Co–**ZPB** and Co–**QDB**, respectively, and 0.1 M for the supporting electrolyte, (n-Bu_4_N)ClO_4_. The electrodes were polished on an MD-Nap polishing pad. A 0.2 M solution of Et_3_NHCl was added via a syringe.

### General Procedure for Hydrogen Production

For photoinduced hydrogen evolution, varying amounts of the catalyst and the 1:1 CH_3_CN/H_2_O solution containing **Fl** and TEA were added in a total volume of 5.0 mL for Co–**ZPB**, and varying amounts of catalyst, **Fl** and TEA in DMF/CH_3_CN/H_2_O (1/4/4) were added to a total volume of 5.0 mL for Co–**QDB**. The pH of this solution was adjusted by adding HCl or NaOH and measured with a pH metre^50^,^51^. Typically, the Co–**ZPB** sample contained Co–**ZPB** (1 × 10^−4^ M), **Fl** (1 × 10^−4^ M) and 5% TEA as the sacrificial electron donor at pH 11.0, and the Co–**QDB** sample contained Co–**QDB** (8 × 10^−5^ M), **Fl** (4 × 10^−5^ M) and 5% TEA as the sacrificial electron donor at pH 10.0. The flask was sealed with a septum, protected from light, and degassed by bubbling nitrogen for 15 min under atmospheric pressure at room temperature. Next, the samples were irradiated by a 500 W xenon lamp; the reaction temperature was maintained at 293 K using a water filter to absorb heat. The generated photoproduct of H_2_ was characterized on a 7890 T GC instrument with a 5 Å molecular sieve column (0.6 m × 3 mm), a thermal conductivity detector, and nitrogen as the carrier gas. The amount of hydrogen generated was determined by the external standard method. The hydrogen in the resulting solution was not measured, and the slight effect of the hydrogen gas generated on the pressure of the Schlenk bottle was neglected in the calculation of the volume of hydrogen gas.

### Synthesis of Co–ZPB

#### Dimethyl 1-benzyl-4-phenyl-1,4-dihydropyridine-3,5-dicarboxylate

Methyl propiolate (1.68 g, 20 mmol), benzaldehyde (1.06 g, 10 mmol), and benzylamine (1.07 g, 10 mmol) in glacial acetic acid (2.0 mL) were heated at 80 °C for 30 min^52^. After cooling, the mixture was poured into water (20 mL) and stirred for 1 h. The solid product was filtered and washed with Et_2_O (3 × 30 mL) to give pure dimethyl 1-benzyl-4-phenyl-1,4-dihydropyridine-3,5-dicarboxylate, which was recrystallized by ethanol (Figure S23 in supporting information). Yield: 1.91 g, 52.3%. ^1^H NMR (400 MHz, DMSO-*d*_6_, ppm): δ 7.51 (s, 2H), 7.45–7.41 (m, 2H), 7.37–7.34 (m, 3H), 7.20–7.16 (m, 2H), 7.13–7.08 (m, 3H), 4.82 (s, 2H), 4.70 (s, 1H), 3.53 (s, 6H).

#### 1-benzyl-4-phenyl-1,4-dihydropyridine-3,5-dicarbohydrazide

A mixture solution of 80% hydrazine hydrate (50 ml) and dimethyl 1-benzyl-4-phenyl-1,4-dihydropyridine-3,5-dicarboxylate (3.63 g, 10 mmol) was stirred at 85 °C over 12 h. The white precipitate was formed, which was collected by filtration, washed with methanol and dried in vacuum. Yield: 1.72 g, 47.3%. ^1^H NMR (400 MHz, DMSO-*d*_6_, ppm): δ 8.67 (s, 2H), 7.42–7.39 (m, 2H), 7.35–7.32 (m, 3H), 7.20 (s, 2H), 7.17–7.12 (m, 4H), 7.09–7.05 (m, 1H), 5.00 (s,1H), 4.60 (s, 2H), 4.12 (s, 4H).

#### Synthesis of H_2_ZPB

1-benzyl-4-phenyl-1,4-dihydropyridine-3,5-dicarbohydrazide (3.63 g, 10 mmol) was added to a ethanol solution (50 mL) containing 2-pyridylaldehyde (2.35 g, 22 mmol). After 5 drops of acetic acid was added, the mixture was heated at 85 °C under magnetic stirring for 12 h according to the ref.^53^. The yellow solid was collected by filtration, washed with methanol and dried in vacuum. Yield: 4.33 g, 79.9%. ^1^H NMR (400 MHz, DMSO-*d*_6_, ppm): δ 11.36 (s, 2H_6_), 8.57 (d, 2H_1_), 8.21 (s, 2H_5_), 7.83–7.78 (m, 4H_3,4_), 7.51–7.47 (m, 2H_2_), 7.46–7.41 (m, 4H_8,11_), 7.39–7.35 (m, 3H_10,12_), 7.27–7.20 (m, 4H_β,γ_), 7.14–7.09 (m, 1H_α_), 5.35 (s, 1H_7_), 4.77 (s, 2H_9_). Anal calc. for C_32_H_27_N_7_O_2_·H_2_O: H 5.22, C 68.68, N 17.52%. Found: H 5.50, C 68.27, N 17.18%. ESI-MS calcd for C_32_H_27_N_7_O_2_ 541.22, found 542.23 [M + H]^+^, 564.21 [M + Na]^+^.

#### Synthesis of Co–ZPB

Co(NO_3_)_2_·6H_2_O (29.1 mg, 0.10 mmol) and H_2_ **ZPB** (54.2 mg, 0.10 mmol) were dissolved in CH_3_OH/CH_2_Cl_3_ (1/1,v/v) to give a red solution. After addition of NaClO_4_, red precipitates formed were isolated and dried under vacuum. Yield: 68%. ^1^H NMR (400 MHz, DMSO-*d*_6_, ppm): δ 11.39 (s, 2H_6_), 8.58 (d, 2H_1_), 8.22 (s, 2H_5_), 7.83 (m, 4H_3,4_), 7.50 (m, 2H_2_), 7.45–7.33 (m, 7H_8,10,11,12_), 7.25 (m, 4H_β,γ_), 7.12 (m, 1H_α_), 5.34 (s, 1H_7_), 4.78 (s, 2H_9_). Anal calc. for Co_3_(C_96_H_79_N_21_O_6_)·3ClO_4_·NO_3_: H 3.69, C 53.38, N 14.27%. Found: H 3.89, C 52.05, N 13.95%. ESI-MS: *m/z*: 947.19 [Co_3_(H**ZPB**)_3_·ClO_4_]^2+^, 996.66 [Co_3_(H**ZPB**)_2_(H_2_ **ZPB**)·2ClO_4_]^2+^.

### Synthesis of Co–QDB

Co(NO_3_)_2_·6H_2_O (30 mg, 0.10 mmol) and H_2_ **QDB** (70 mg, 0.10 mmol) were dissolved in DMF and then stirred for 2 h. The solution was left for several days at room temperature to give X-ray quality black block crystals. Yield: 65%. ^1^H NMR (400 MHz, DMSO-*d*_6_, ppm): δ 12.24 (s, 2H), 8.61 (s, 2H), 8.46 (m, 2H), 8.12 (m, 2H), 8.05 (m, 4H), 7.79 (m, 2H), 7.76 (s, 1H), 7.66 (m, 2H), 7.46 (s, 2H), 7.40–7.27 (m, 10H), 4.85 (s, 4H). Anal calc. for Co_3_(C_126_H_95_N_21_O_6_)·2PF_6_: H 3.88, C 61.37, N 11.93%. Found: H 4.08, C 60.06, N 11.69%. ESI-MS: *m/z*: 1088.05 [Co_3_(**QDB**)(H**QDB**)_2_]^2+^, 1119.55[Co_3_(H**QDB**)_3_·NO_3_]^2+^, 1161.03 [Co_3_(H**QDB**)_3_ PF_6_]^2+^.

## Electronic supplementary material

Supplementary Information
