# Supplementary material for: DHPA-Containing Cobalt-Based Redox Metal-Organic Cyclohelicates as Enzymatic Molecular Flasks for Light-Driven H2 Production
Source: Sci Rep. 2017 Oct 30;7:14347. doi: 10.1038/s41598-017-14728-8 (PMC5662590; doi:10.1038/s41598-017-14728-8)
Supplement: Supplementary file 1 — Supplementary Information [file 41598_2017_14728_MOESM1_ESM.pdf]

Supplementary Information

DHPA-Containing Cobalt-Based Redox Metal-Organic Cyclohelicates as Enzymatic Molecular Flasks for Light-Driven H_2 Production

Liang Zhao, Jian Wang, Pengyan Wu, Cheng He, Xiangyang Guo & Chunying Duan*

State Key Laboratory of Fine Chemicals, Dalian University of Technology, Dalian, 116012, P. R. China

1. Single Crystal X-ray Crystallography.

Intensities of the Co–**QDB** was collected on a Bruker SMART APEX CCD diffractometer with graphite-monochromated Mo-Kα (λ = 0.71073 Å) using the SMART and SAINT programs[S1,S2]. The structures were solved by direct methods and refined on F^2 by full-matrix least-squares methods with SHELXTL *version* 5.1. Crystal data of Co–**QDB**: $Co_3(C_{126}H_{97}N_{21}O_6)\cdot 10CH_3OH\cdot 2.9H_2O\cdot 2PF_6$, M = 2838.63, Triclinic, space group P-1, red block, a = 18.4221 (15), b = 18.4307 (14), c = 28.060 (2) Å, α = 93.188 (5), β = 94.619 (6), γ = 119.805 (5), V = 8189.6 (11) Å^3, Z = 2, Dc = 1.151 g cm^{-3}, μ(Mo-Kα) = 0. 394 mm^{-1}, T = 200(2) K. 25502 unique reflections [R_{int} = 0.1418]. Final R_1 [with $I > 2\sigma(I)$] = 0.1050, wR_2 (all data) = 0.2664. CCDC number 1029242.

In the structural refinements of Co–**QDB**, except some solvent molecules, the skeleton non-hydrogen atoms were refined anisotropically. Hydrogen atoms within the ligand backbones were fixed geometrically at calculated distances and allowed to ride on the parent non-hydrogen atoms. To assist the stability of refinements, several restrains were applied: Two of the six bezene rings in the benzylamine groups were disordered into two parts with the *s.o.f* of each part being fixed at 0.5. Three of the six bezene rings in the benzylamine groups were restrained as idealized geometry. Four of the six fluoride atoms on the one of the three PF_6^- anions were disordered into two parts with free values and another of the three PF_6^- anions was disordered into two parts with free values. Two PF_6^- anions and many methanol molecules were restrained as idealized geometry. Thermal parameters on adjacent atoms in two of the three PF_6^- anion and the bezene rings of the benzylamine groups were restrained to be similar.

1.1 Figure S1 An ORTEP plot of the molecular tetrahedron Co–**QDB**, showing 30% probability displacement ellipsoids of non-hydrogen atoms. Hydrogen atoms are omitted for clarity.

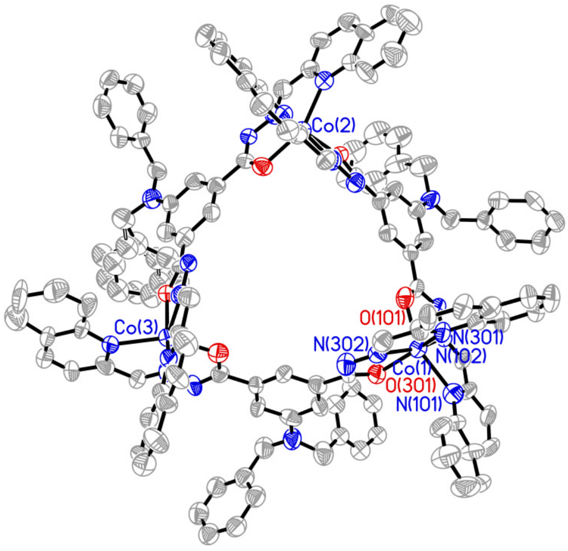

1.2 Table S1 Selective bond distance (Å) in Co–**QDB**.

	bond distance (Å)		bond distance (Å)
Co(1)-N(102)	2.033(7)	C(115)-O(102)	1.260(11)
Co(1)-N(302)	2.035(8)	C(115)-N(106)	1.409(12)
Co(1)-O(101)	2.065(6)	C(116)-N(105)	1.228(12)
Co(1)-N(301)	2.170(8)	C(117)-N(104)	1.352(12)
Co(1)-O(301)	2.177(7)	C(125)-N(104)	1.362(12)
Co(1)-N(101)	2.207(9)	C(128)-N(107)	1.345(11)
C(101)-N(101)	1.328(12)	C(129)-N(107)	1.425(13)
C(109)-N(101)	1.289(12)	C(136)-N(107)	1.498(13)
C(110)-N(102)	1.266(11)	N(102)-N(103)	1.378(10)
C(111)-O(101)	1.282(11)	N(105)-N(106)	1.385(9)
C(111)-N(103)	1.340(12)		

1.3 Table S2 Selective bond angle (°) in Co–**QDB**.

	bond angle (°)		bond angle (°)
N(102)-Co(1)-N(302)	161.5(3)	O(201)-Co(2)-N(104)	89.9(3)
N(102)-Co(1)-O(101)	75.6(3)	O(102)-Co(2)-N(104)	149.4(3)
N(302)-Co(1)-O(101)	92.1(3)	N(202)-Co(2)-N(201)	76.3(3)
N(102)-Co(1)-N(301)	117.0(3)	N(105)-Co(2)-N(201)	116.0(3)
N(302)-Co(1)-N(301)	76.0(3)	O(201)-Co(2)-N(201)	150.5(3)
O(101)-Co(1)-N(301)	89.8(3)	O(102)-Co(2)-N(201)	90.4(3)
N(102)-Co(1)-O(301)	92.9(3)	N(104)-Co(2)-N(201)	95.9(3)
N(302)-Co(1)-O(301)	75.2(3)	N(205)-Co(3)-N(305)	161.3(3)
O(101)-Co(1)-O(301)	99.2(3)	N(205)-Co(3)-O(302)	92.9(3)
N(301)-Co(1)-O(301)	150.1(3)	N(305)-Co(3)-O(302)	75.3(3)
N(102)-Co(1)-N(101)	76.1(3)	N(205)-Co(3)-N(204)	76.3(3)
N(302)-Co(1)-N(101)	117.6(3)	N(305)-Co(3)-N(204)	117.6(3)
O(101)-Co(1)-N(101)	150.2(3)	O(302)-Co(3)-N(204)	90.7(3)
N(301)-Co(1)-N(101)	94.9(3)	N(205)-Co(3)-N(304)	116.3(3)
O(301)-Co(1)-N(101)	91.3(3)	N(305)-Co(3)-N(304)	76.3(3)
N(202)-Co(2)-N(105)	162.8(4)	O(302)-Co(3)-N(304)	150.7(3)
N(202)-Co(2)-O(201)	75.3(3)	N(204)-Co(3)-N(304)	96.2(3)
N(105)-Co(2)-O(201)	93.4(3)	N(205)-Co(3)-O(202)	73.5(3)
N(202)-Co(2)-O(102)	93.7(3)	N(305)-Co(3)-O(202)	93.5(3)
N(105)-Co(2)-O(102)	74.9(3)	O(302)-Co(3)-O(202)	98.0(2)
O(201)-Co(2)-O(102)	99.2(3)	N(204)-Co(3)-O(202)	148.9(3)
N(202)-Co(2)-N(104)	116.8(3)	N(304)-Co(3)-O(202)	90.6(3)
N(105)-Co(2)-N(104)	75.4(3)		

2. Figures

Figure S2 ESI-MS spectrum of the system containing the Co−**ZPB** (0.1 mM), fluorescein (0.1 mM) and TEA (5%) after reaction 7 hours.

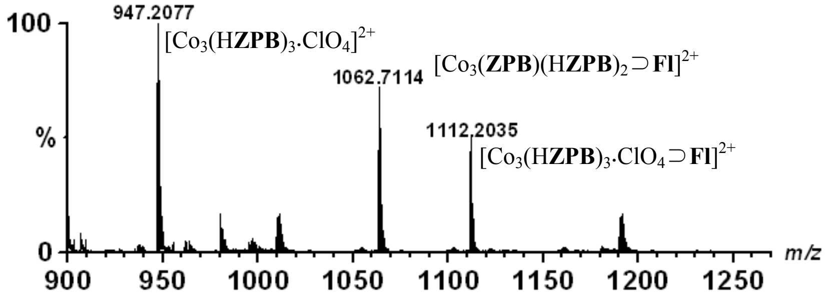

Figure S3 ESI-MS of (a) Co–**QDB** (0.1 mM), (b) Co–**QDB** upon addition of 0.5 equiv of fluorescein and (c) Co–**QDB** (0.1 mM) upon addition of 1 equiv of GSH in DMF/CH_3OH solution.

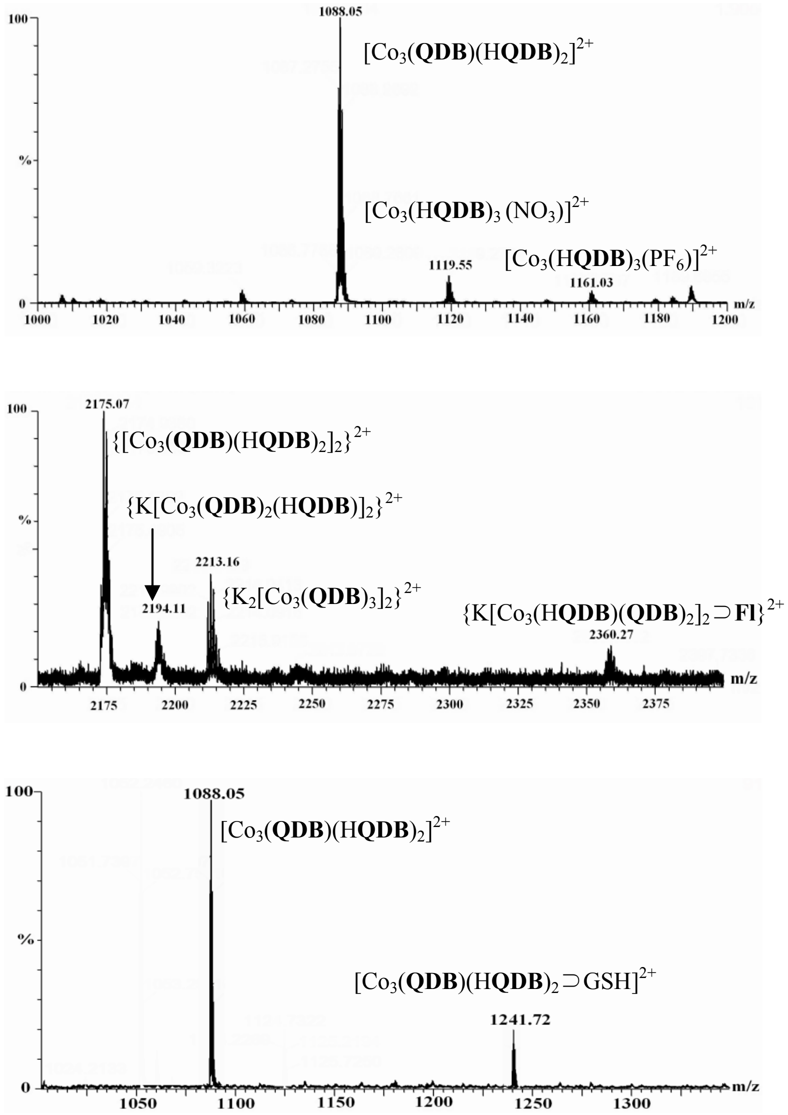

Figure S4 ESI-MS spectrum of the system containing the Co−**QDB** (0.08 mM), fluorescein (0.04 mM) and TEA (5%) after reaction 6 hours.

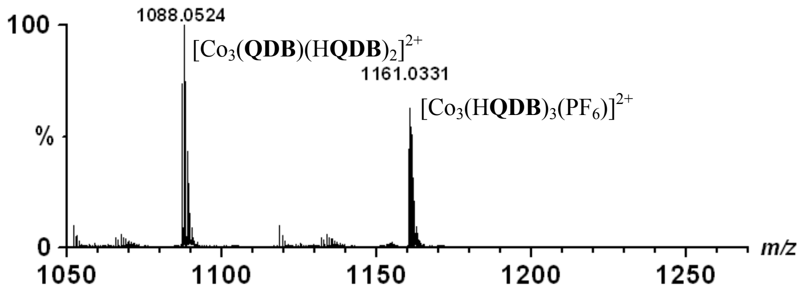

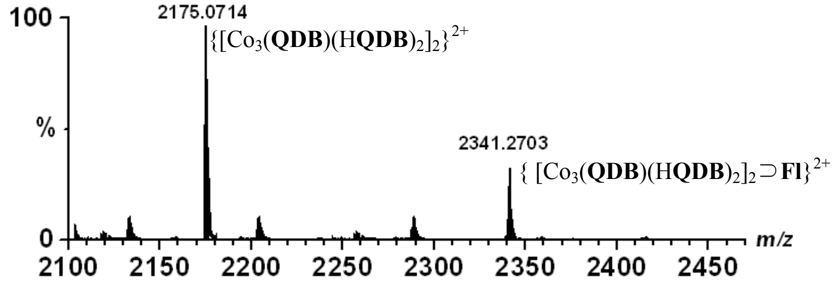

Figure S5 Partial ^{1}H NMR spectra of Co–**ZPB** (5 mM) (top); of Co–**ZPB** with fluorescein (5 mM) (middle) and fluorescein itself (bottom) in DMSO-d_6, showing the chemical shift of the fluorescein within the cavity.

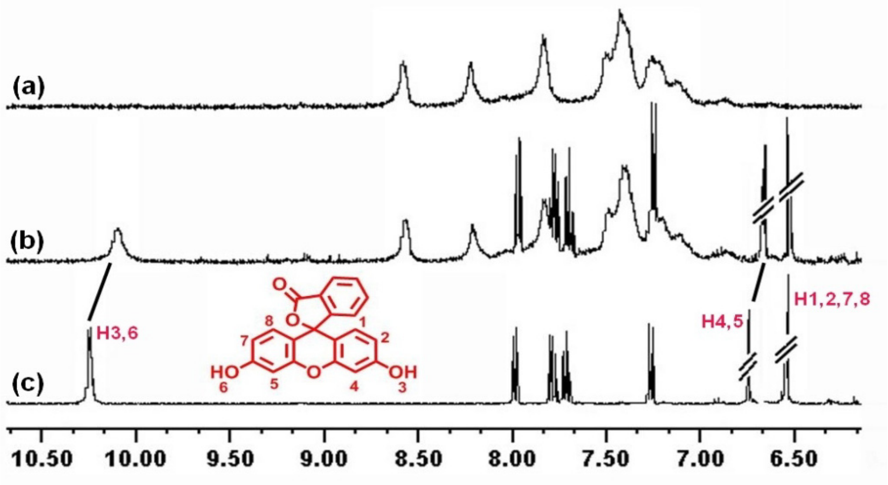

Figure S6 Partial ^{1}H NMR spectra (aromatic region) of free Co-**ZPB** (5 mM) (a), free ATP (5 mM) (c) and of Co-**ZPB** with ATP (b) in a 1:2 molar ratio in d_6-DMSO/D_2O, showing the chemical shift of the ATP within the cavity.

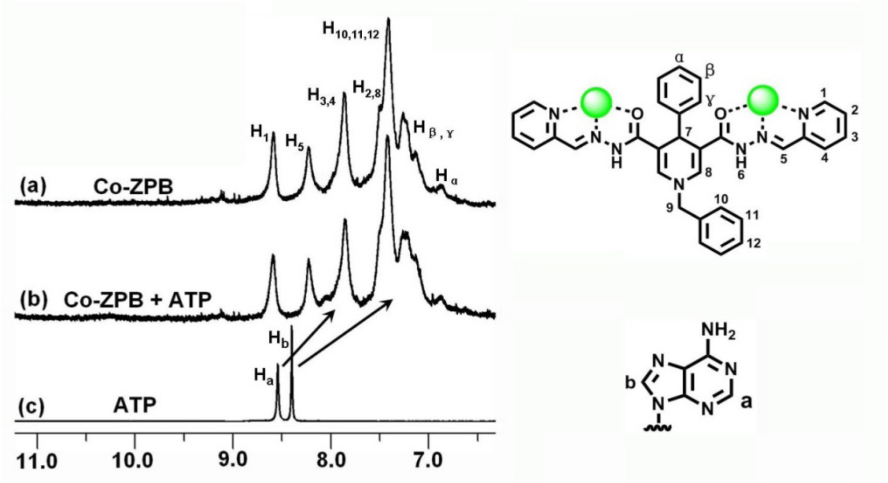

Figure S7 Partial ^{1}H NMR spectra of Co–**QDB** (5 mM) (top); of Co–**QDB** with fluorescein (2.5 mM) (middle) and fluorescein itself (bottom) in DMSO-d_6, showing the chemical shift of the fluorescein within the cavity.

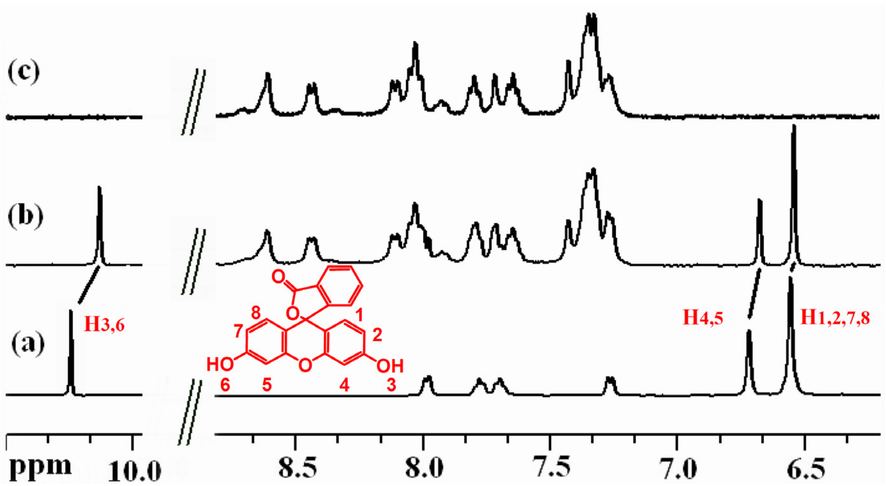

Figure S8 ^{1}H NMR spectra of Zn–**PDB** (10 mM) (top); of Zn–**PDB** with GSH (10 mM) (middle) and GSH itself (bottom) in DMSO-d_6, showing the chemical shift of the GSH within the cavity. [S3]

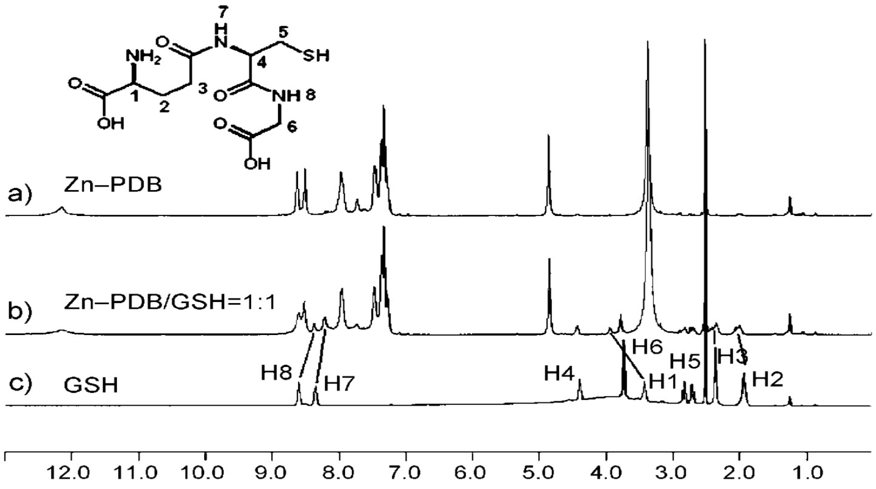

Figure S9 Family of UV-vis spectrum of Co-**ZPB** (10 μM in CH_3CN/H_2O = 1/1) upon the addition of fluorescein.

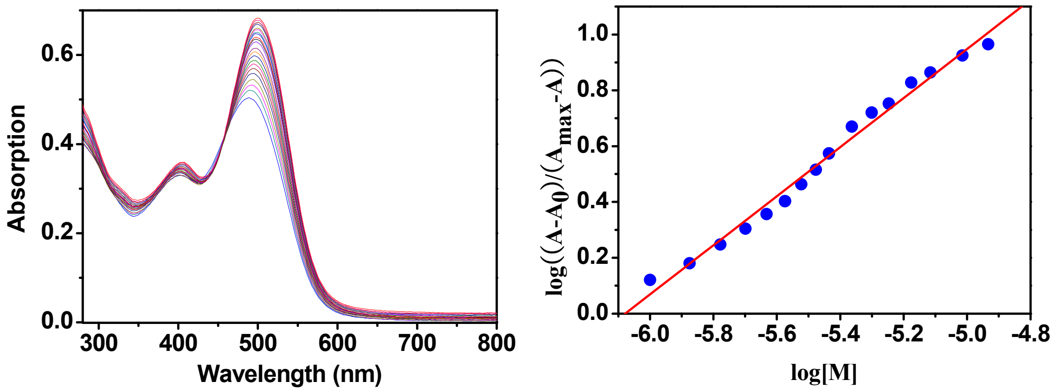

Figure S10 Family of UV-vis spectrum of Co-**QDB** (10 μM in CH_3CN/H_2O = 1/1) upon the addition of fluorescein.

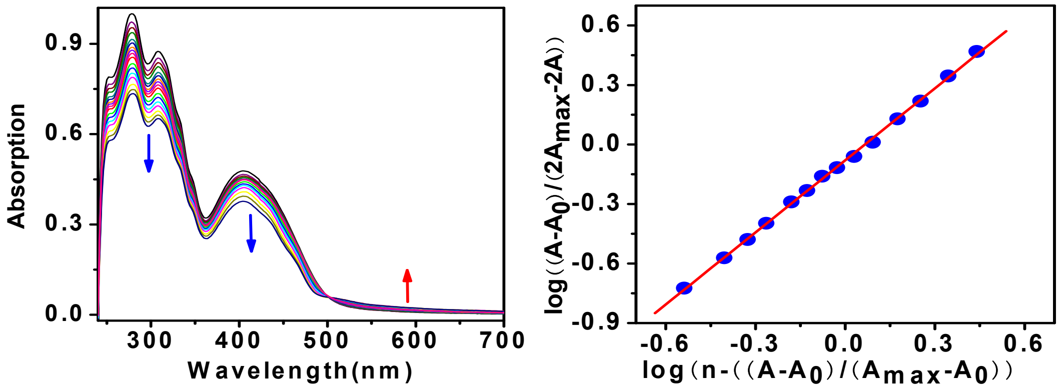

Figure S11 Family of fluorescence of fluorescein (10 μM) in 1:1 CH_3CN/H_2O at pH 11.0 upon addition of TEA up to 500 mM, respectively.

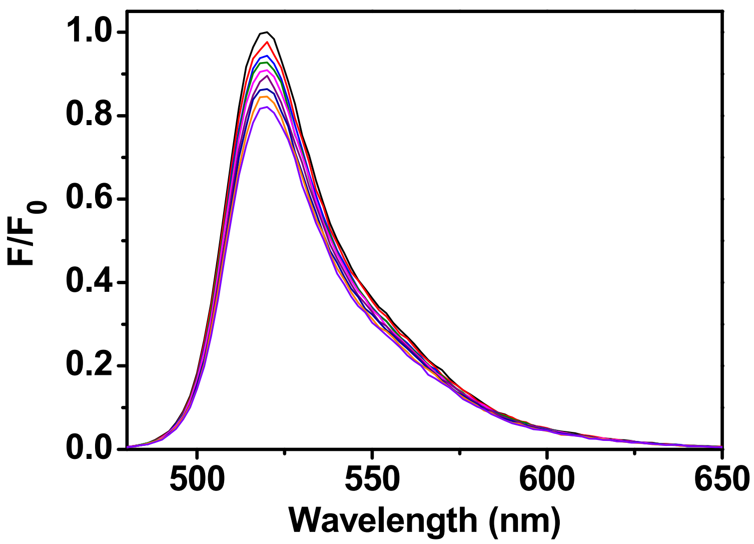

Figure S12 Family of fluorescence of fluorescein (10 μM) in 1:1 CH_3CN/H_2O at pH 11.0 upon addition of Co-**ZPB** up to 3.0 μM, respectively.

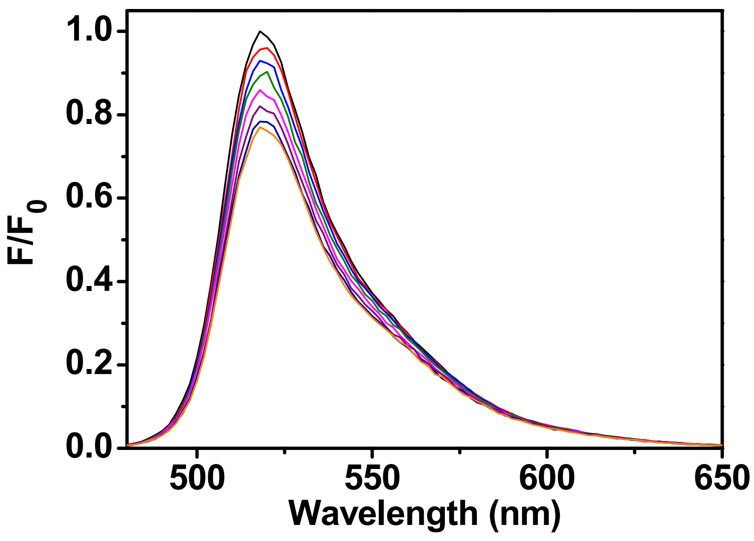

Figure S13 Family of fluorescence of fluorescein (10 μM) in 1:1 CH_3CN/H_2O at pH 10.0 upon addition of TEA up to 500 mM, respectively.

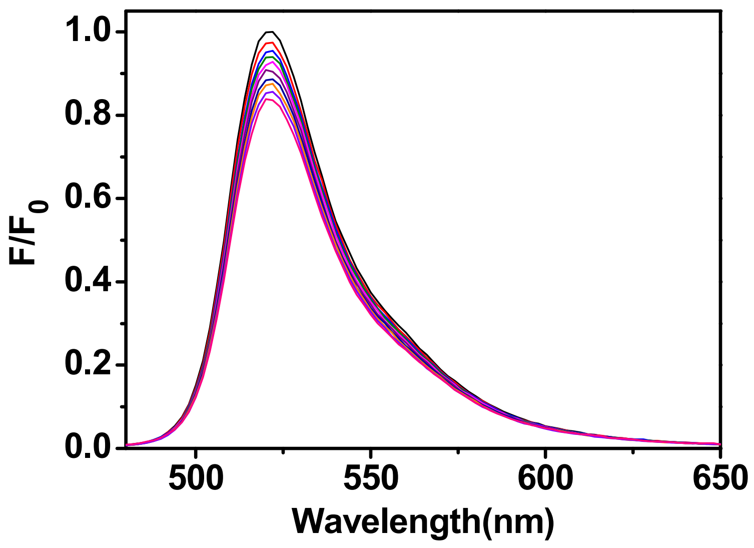

Figure S14 Family of fluorescence of fluorescein (10 μM) in 1:1 CH_3CN/H_2O at pH 10.0 upon addition of Co−**QDB** up to 3.0 μM, respectively.

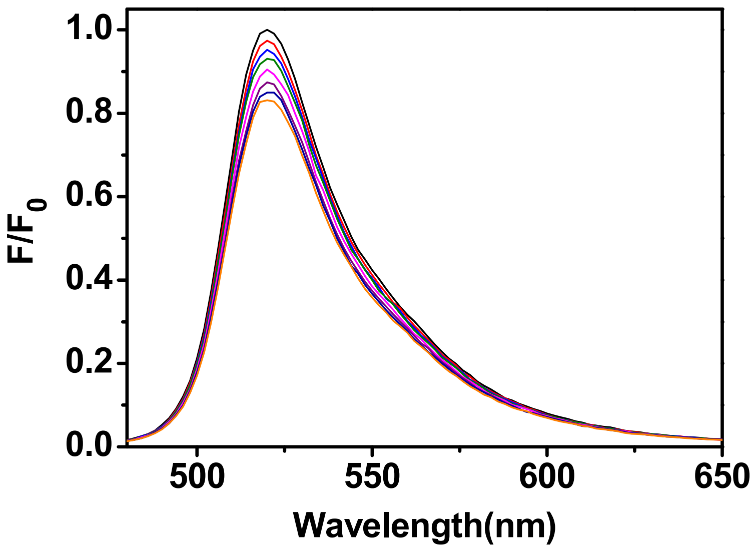

Figure S15 Influence of the pH on photoinduced hydrogen production containing Co–**ZPB** (1×10^{-4} M), **Fl** (1×10^{-4} M), TEA 5%, in CH_3CN/H_2O=1/1 in 6 h of irradiation and hydrogen production from systems at pH 11.0 upon irradiation with different concentration.

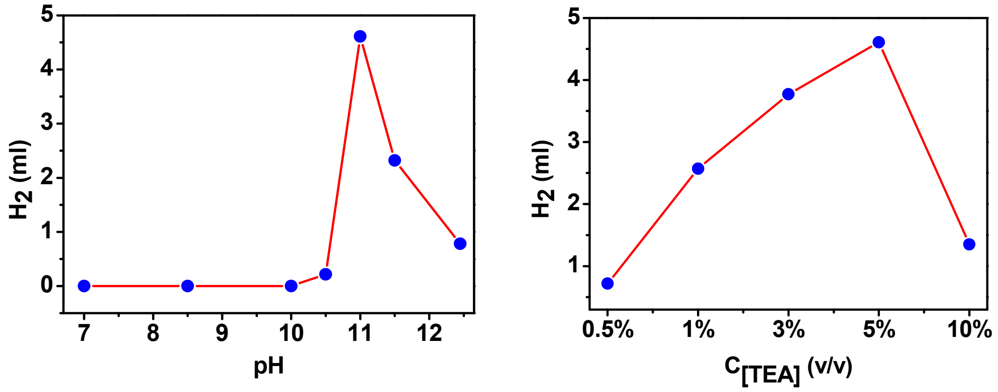

Figure S16 Influence of the pH on photoinduced hydrogen production containing Co–**QDB** (8×10^{-5} M), **Fl** (4×10^{-5} M), TEA 5%, in $CH_3CN/DMF/H_2O$=4/1/4 in 6 h of irradiation and hydrogen production from systems at pH 10.0 upon irradiation with different concentration.

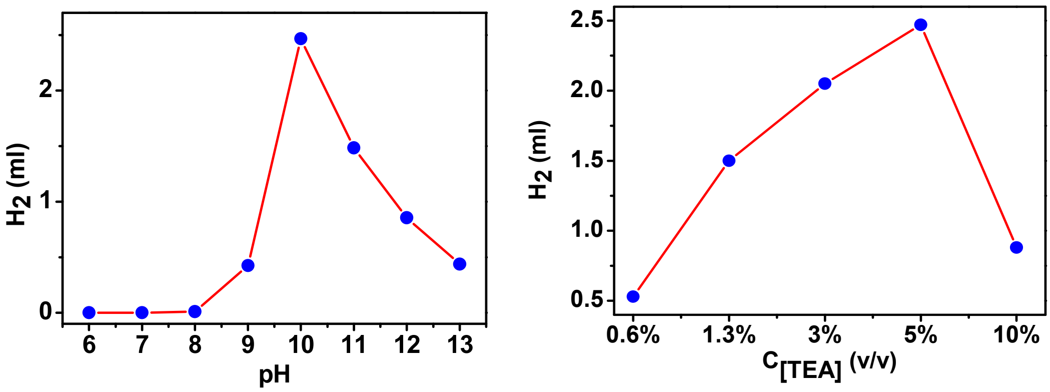

Figure S17 Family of UV-vis spectrum of Co–**ZPB** (10 μM in CH_3CN/H_2O = 1/1) upon the addition of 100 μM ATP.

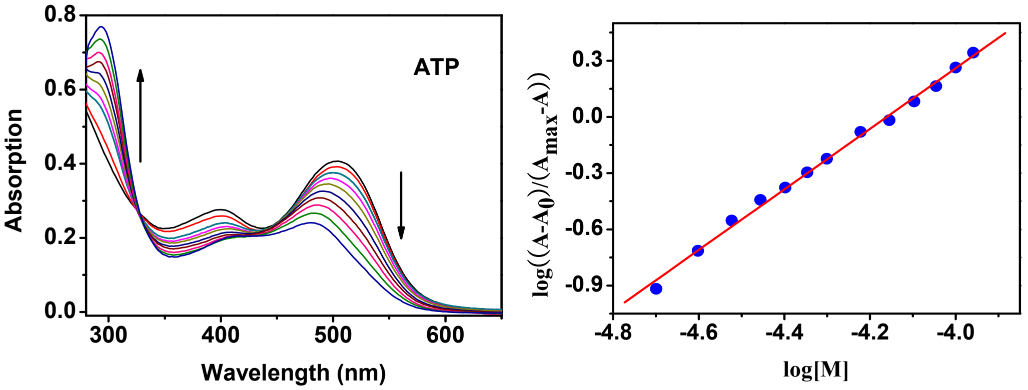

Figure S18 Family of UV-vis spectrum of Co–**QDB** (10 μM in CH_3CN/H_2O = 1/1) upon the addition of 50 μM GSH.

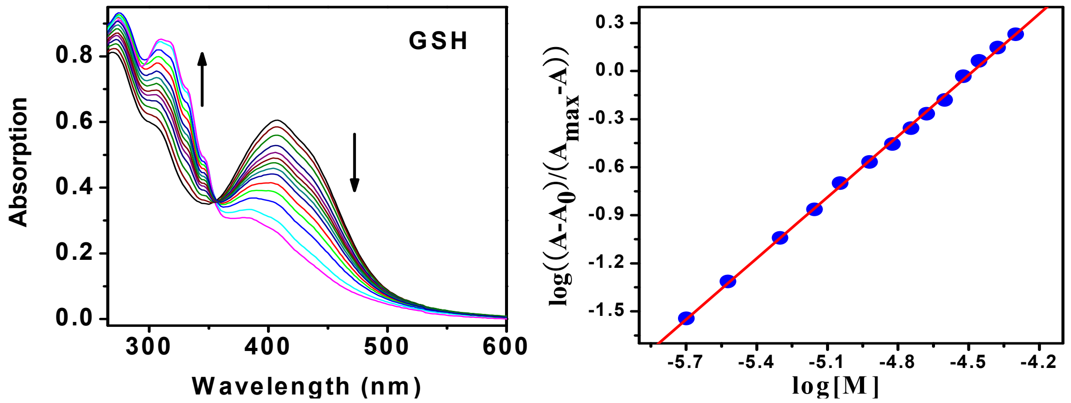

Figure S19 H_2 production after irradiation of the system containing TEA (5%), Fl (0.1 mM) and different concentrations of Co–**ZPB** (1:1 and 0.5:1 ***vs.* Fl**).

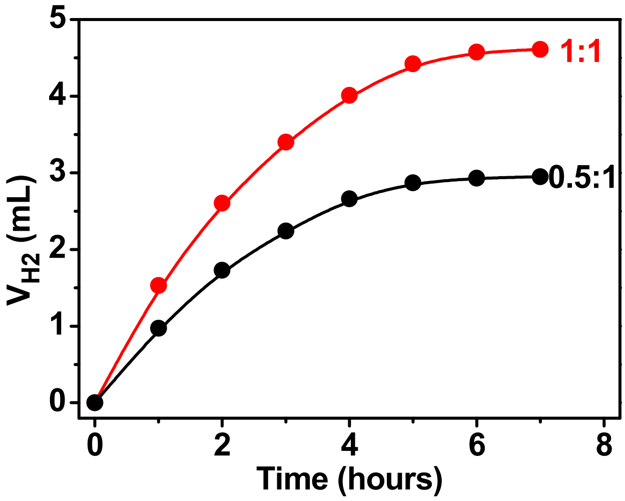

Figure S20 Cyclic voltammograms of Co−**ZPB** (0.1 mM) and H_2**ZPB** (0.3 mM) containing *n*-Bu_4NClO_4 (0.1 M) in CH_3CN and DMF solution, respectively. Scan Rate: 100 mV/s..

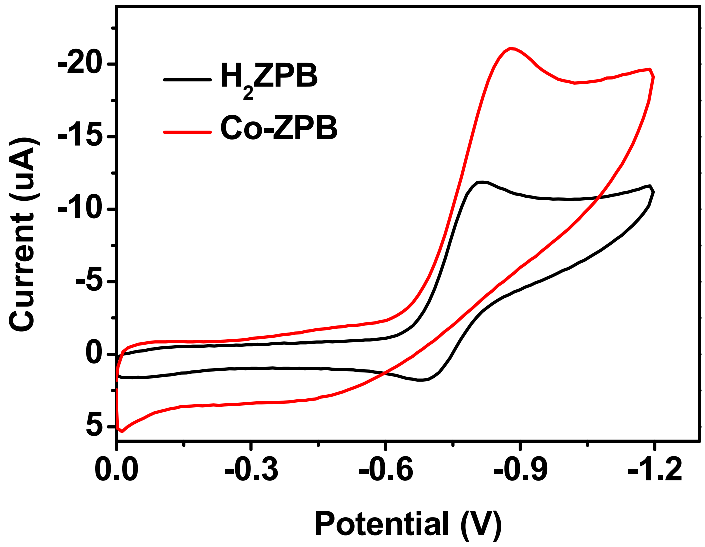

Figure S21 Cyclic voltammograms of Co−**QDB** (1.0 mM) containing *n*-Bu_4NClO_4 (0.1 M) in a DMF solution and upon the addition of Et_3NHCl with concentrations of 0.4 mM (red line), 0.8 mM (blue line), 1.2 mM (green line), 1.6 mM (cyan line), 2.0 mM (magenta line), and 2.4 mM (navy line), respectively. Scan Rate: 100 mV/s. The inset showing the i_c *vs.* [$HNEt_3$].

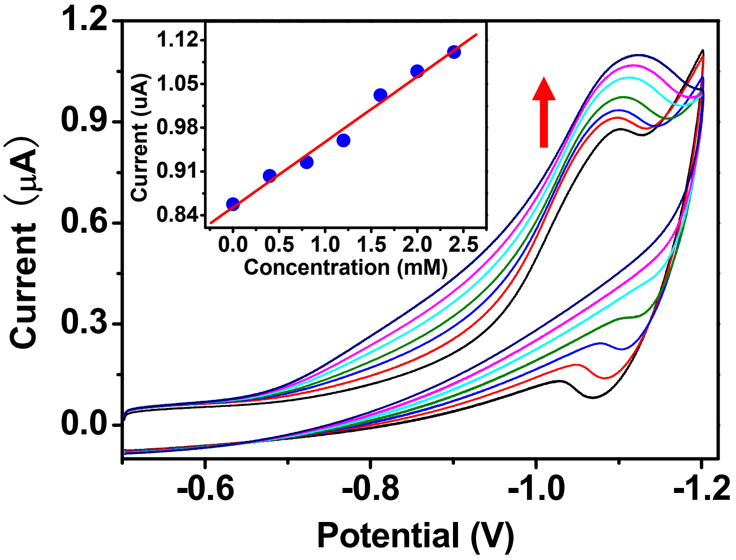

Figure S22 Cyclic voltammograms of H_2**QDB** (3.0 mM) containing *n*-Bu_4NClO_4 (0.1 M) in a DMF solution. Scan Rate: 100 mV/s.

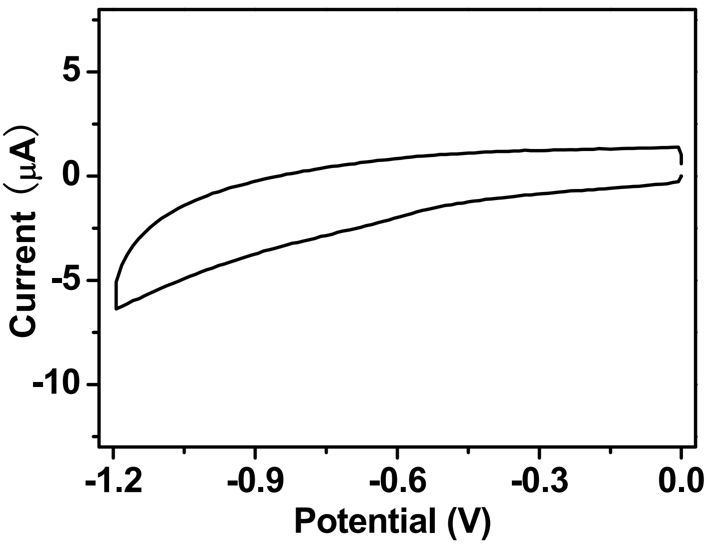

Figure S23 The synthetic routes of the H_2**ZPB**.

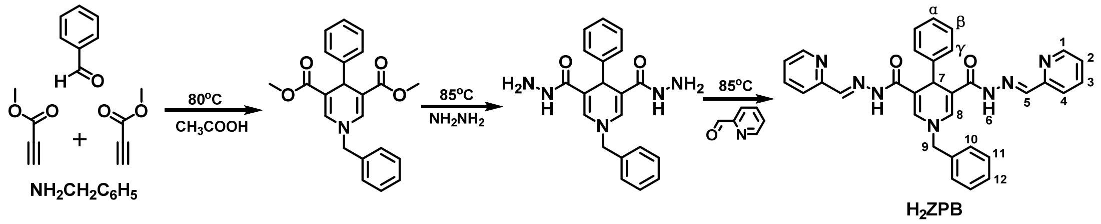

Reference.

S1: SMART, Data collection software (version 5.629) (Bruker AXS Inc.; Madison, WI, 2003).

S2: SAINT, Data reduction software (version 6.45) (Bruker AXS Inc.; Madison, WI, 2003).

S3: Wang, J. Wu, H. M. He, C. Zhao, L. & Duan, C. Y. Metal–Organic Cyclohelicates as Optical Receptors for Glutathione: Syntheses, Structures, and Host–Guest Behaviors. *Chem. Asian J.*, **6**, 1225–1233 (2011).
